# How Study Environments Foster Academic Procrastination: Overview and Recommendations

**DOI:** 10.3389/fpsyg.2020.540910

**Published:** 2020-11-02

**Authors:** Frode Svartdal, Tove I. Dahl, Thor Gamst-Klaussen, Markus Koppenborg, Katrin B. Klingsieck

**Affiliations:** ^1^Department of Psychology, UiT The Arctic University of Norway, Tromsø, Norway; ^2^Evaluation of Studies and Teaching and Higher Education Research, University of Cologne, Cologne, Germany; ^3^Department of Psychology, Paderborn University, Paderborn, Germany

**Keywords:** academic procrastination, study environments, social factors, self-regulation, impulsivity, task aversiveness

## Abstract

Procrastination is common among students, with prevalence estimates double or even triple those of the working population. This inflated prevalence indicates that the academic environment may appear as “procrastination friendly” to students. In the present paper, we identify social, cultural, organizational, and contextual factors that may foster or facilitate procrastination (such as large degree of freedom in the study situation, long deadlines, and temptations and distractions), document their research basis, and provide recommendations for changes in these factors to reduce and prevent procrastination. We argue that increased attention to such procrastination-friendly factors in academic environments is important and that relatively minor measures to reduce their detrimental effects may have substantial benefits for students, institutions, and society.

Procrastination, voluntarily delaying tasks despite expecting to be worse off (Steel, 2007), is common among students. Conservative estimates indicate that at least half of all students habitually procrastinate tasks that are important to them, such as reading for exams, writing term papers, and keeping up with weekly assignments (Solomon and Rothblum, 1984; Tice and Baumeister, 1997; Pychyl et al., 2000; Schouwenburg, 2004; Steel, 2007). Consequences are negative, both for academic performance and retention (Ellis and Knaus, 1977; Klassen et al., 2008; Zarick and Stonebraker, 2009; Grau and Minguillon, 2013; Kim and Seo, 2015) as well as for health and well-being (Flett et al., 1995; Tice and Baumeister, 1997; Stöber and Joormann, 2001; Sirois, 2014).

Despite the possibility that academic environments may contribute significantly to this situation, the majority of research efforts to clarify mechanisms involved in procrastination has focused on individual variables related to personality, motivation, affect, and others (for reviews, see van Eerde, 2003; Steel, 2007; Klingsieck, 2013). The present paper takes a different view, focusing on situational, social, contextual, cultural, and organizational factors common in academic environments. Based on the procrastination literature, we present a selection of such factors and show how they increase the probability of procrastination. Negative effects may be general in that most students suffer. Often, however, “procrastination-friendly” factors may also affect students differentially, those being prone to procrastination in the first place being particularly vulnerable (e.g., Nordby et al., 2017; Visser et al., 2018). Thus, ideas on how to address these factors to make the academic environment more “procrastination-*un*friendly” are important.

We identify nine broad factors known to increase procrastination. The factors selected serve as important examples rather than an exhaustive list. For each factor, we link it to common features of academic environments, providing examples and other forms of documentation to demonstrate its significance in facilitating procrastination. We then formulate specific advice on how the negative influence of each factor may be alleviated or remedied by relatively simple structural, organizational, and educational measures.

## Characteristics of Academic Procrastination

Academic procrastination occurs when a student delays work related to academic tasks (Solomon and Rothblum, 1984; Tice and Baumeister, 1997; Pychyl et al., 2000; Schouwenburg, 2004; Steel, 2007). For such delays to be regarded as procrastination, the student voluntarily chooses to delay despite expecting to be worse off (Steel, 2007). Thus, there is an important distinction between delays that are sensible and rational (e.g., “I chose to postpone my thesis submission because my supervisor advised me to revise the discussion part”) and those that are not (e.g., “I did not prepare for the seminar today, I watched a movie instead”). In effect, academic procrastination is a form of irrational delay, as the person acts against better judgment.

The delays seen in academic procrastination may result from late onset (e.g., “I did not start writing until just one week before deadline”) and impulsive diversions during work (e.g., “I was working, but got tired and had a coffee with a friend instead”) (Svartdal et al., 2020). As is well documented in the research literature over the past 40 years, such delays and diversions are related to personality factors, as for example impulsiveness and a preference for short-term gratification, deficiencies in planning and self-regulation, low self-efficacy, tiredness, and low energy, and task avoidance (van Eerde, 2000; Steel, 2007; Steel et al., 2018). The majority of this research has been correlational. Because procrastination is a complex phenomenon unfolding over time and in interaction with situational, social, contextual, cultural, and organizational factors, it is important also to focus on exogenous factors involved in this complex and dynamic phenomenon. The relative lack of such studies is unfortunate and clearly represents a gap in the procrastination field. We argue that this is particularly unfortunate in the academic area, as the student is confronted with situational, social, contextual, cultural, and organizational factors that are prone to instigate and maintain procrastination in tasks that constitute core student activities.

### How Is Academic Procrastination Measured?

Academic procrastination is typically measured with self-report tools, as is general procrastination. In measuring academic procrastination, some scales focus on general tendencies to delay tasks unnecessarily, with few if any items covering academic tasks specifically. For example, the General Procrastination Scale (20 items; Lay, 1986), academic version, has 16 items common with the general version and four items addressing academic tasks specifically (e.g., Item 2, “I do not do assignments until just before they are to be handed in”). Similarly, the Tuckman procrastination scale (16 items; Tuckman, 1991) measures academic procrastination solely by general items (e.g., item 1 “I needlessly delay finishing jobs, even when they’re important”). Other academic procrastination scales focus on academic tasks exclusively, such as the Academic Procrastination State Inventory (APSI; Schouwenburg, 1995) and the Procrastination Assessment Scale (PASS; Solomon and Rothblum, 1984). The PASS contains 44 questions that address various forms of academic tasks (e.g., studying for an exam, writing a term paper) in terms of how often they are procrastinated, to which extent such procrastination represents a problem, and willingness to change.

Importantly, scores on academic procrastination scales have been validated against procrastination in real academic tasks. For example, Tuckman compared scores on his scale against actual performance points on voluntary homework assignments, where students had the opportunity to write and submit written material to gain extra course credits. He found a negative correlation, *r* =−0.54, between these measures, concluding that “students are well aware of their own tendencies and can report them with great accuracy” (p. 9). More recent findings (e.g., Tice and Baumeister, 1997; Steel et al., 2018) confirm a relatively close correspondence between students’ self-reported procrastination and relevant behavioral measures.

### Detrimental Effects of Academic Procrastination

It is important to recognize that procrastination is not only an issue related to effective academic work. Although performance (grades) is negatively related to procrastination (for review, see Kim and Seo, 2015), other important problems associated with procrastination are stress, reduced well-being, and mental and physical health problems (e.g., Tice and Baumeister, 1997). For academic procrastination, the increased stress associated with procrastination seems to be important (e.g., Sirois, 2007, 2014). Recognition of the procrastination problem as a health issue, as well as a performance issue, is imperative. In Norway, as well as in other European countries, surveys of student health indicate that an increasing number of students report psychological problems, often of serious nature. For example, in a large-scale survey among Norwegian students, the Students’ Health and Wellbeing Study (Knapstad et al., 2018; *N* = 50,000), 29% of all students reported serious psychological problems. We do not know the role of procrastination in this situation, but it is likely that procrastination may be a contributing factor as well as a consequence. Hence, the role of the environmental factors in encouraging procrastinating is important to assess from a health perspective also.

## Social and Contextual Factors Facilitating Procrastination

### Rationale for Selection of Factors

In the sections to come, we address situational, social, contextual, cultural, and organizational factors that are documented as facilitators of procrastination. In selection of factors, the authors first discussed a larger pool of factors and evaluated their relation to the academic situation. Then, based on expert judgment, we selected nine factors that met the following criteria: They (a) reflect well-documented research findings in the procrastination field; (b) represent factors present in the academic situation beyond the student’s control (e.g., long deadlines), or factors that cannot easily be remedied by the student independently of educational, social, or organizational measures (e.g., task aversion); and that (c) measures taken to change the factor is likely to reduce procrastination. The discussion of each factor is not intended as a complete review, as a review at this stage of research would be premature. Rather, for each factor, we highlight central findings connecting the factor to procrastination research, its relation to the academic environment, and remedies that may alleviate the detrimental effects associated with a given factor. Table 1 presents an overview of the factors discussed.

**TABLE 1:** Factors reliably associated with procrastination, and their relation to the study environment.

1.Large degree of freedom in the study situation	Procrastination is regarded as a self-regulation failure, making procrastinators vulnerable when working under unstructured conditions
2.Long deadlines	Procrastination is more likely to occur if the outcome of an activity offers rewards in the distant future, making long deadlines a factor that fosters procrastination
3.Task aversiveness	Bad mood and negative feelings associated with aversive tasks are repaired by avoiding the task and engaging in a more pleasant task instead
4.Temptations and distractions	People are tuned toward attainment of positive outcomes and escape/avoidance from aversive events. In procrastinators, this picture is exaggerated, with current attractive and aversive events dominating over distant ones.
5.Limited information for proper self-monitoring	The study environment does not provide reliable information for the student to manage attention toward own behavior and performance, increasing the risk of self-regulation failure
6.Low focus on study skills training	Lack of study skills is often reported as a main reason for academic procrastination, but academic institutions often do not provide effective study skills training
7.Lack of efficacy-building opportunities	Self-efficacy is an important determinant of academic performance. With limited opportunity to build self-efficacy in the academic environment, the likelihood of procrastination increases.
8.Ineffective group work	Students participate in group work, often lacking skills necessary to succeed. Evidence suggests that group work with interdependence may be associated with reduced procrastination.
9.Influence of peers	Social norms can reduce procrastination when these norms imply beginning a task on time; observational learning may influence students’ self-regulatory skills

Note that the factors are quite heterogeneous. Some factors (e.g., large degree of freedom in the study situation, long deadlines) identify organizational and structural properties of the academic environment, whereas others emphasize subjective evaluations (e.g., task aversiveness). Also note that the factors discussed may demonstrate “main effects” as most students may be affected, as well as interactive effects where individual characteristics act as moderators. For example, temptations and distractions in the academic environment may be detrimental for most students, but particularly so for individuals high in impulsivity and distractibility (e.g., Steel et al., 2018). Furthermore, the order of factors discussed does not indicate differences in importance. In fact, the effect sizes associated with each factor may be difficult to quantify in academic contexts. Finally, a caution on the use of the term “factor.” We use this term to denote facets or variables in the academic settings that identify features known to relate strongly to procrastination. As these are exogenous factors in the procrastination equation, they represent potential conditions that can be altered in order to affect the probability of procrastination. In the present context, we do not make strong assumptions about causality; rather, we argue that such potential causal relations should receive increased attention in future research.

### Large Degree of Freedom in the Study Situation

#### Relevant Research

In his comprehensive review of research on procrastination, Steel (2007) coined procrastination a quintessential self-regulatory failure. Procrastinators are present-oriented and impulsive and tend to score low on tests measuring conscientiousness and planning, and high on susceptibility to temptation (Lay and Schouwenburg, 1993; van Eerde, 2003; Steel, 2010). Procrastinators make plans, only to reverse them when encountering distractions and temptations during goal implementation (Steel et al., 2018). Hence, procrastinators are particularly vulnerable when working under unstructured conditions and when long-term plans are delegated to the individual.

#### Relation to the Academic Environment

Results from qualitative studies exemplify the negative role of freedom in the study situation in several ways, as too little regulations in studies (Grunschel et al., 2013), low degree of external structure (Klingsieck et al., 2013), or insufficient direction of lecturers (Patrzek et al., 2012). Overall, students reported feeling lost and overwhelmed by the task of planning a whole course of studies, a semester, or even an exam phase on their own. Thus, students lacking self-management skills such as planning and prioritizing tasks (e.g., Lay and Schouwenburg, 1993) and metacognitive learning strategies (e.g., Wolters, 2003; Howell and Watson, 2007) should feel particularly lost when facing a situation with a large degree of freedom. The autonomy associated with a large degree of freedom in the study situation makes the student particularly vulnerable if skills are low (→Low focus on study skills training) and if the student fails to develop good habits and routines. Habits help people accomplish more and procrastinate less (e.g., Steel et al., 2018). Of note, study topics may vary in how much freedom they offer to the student. Some study programs are strictly structured and may even involve a common study group from start to finish (e.g., medicine), whereas other study topics are less structured and may also, by the nature of their contents, appear as more “procrastination friendly” (e.g., Nordby et al., 2017).

#### Remedies

While direct procrastination prevention and intervention programs train the self-management skill of students (for a summary, see van Eerde and Klingsieck, 2018), remedies should also be implemented on the level of study programs and the level of courses. Especially for beginning students, unnecessary options present opportunities for students to procrastinate and should be accompanied by remedial measures. For example, Ariely and Wertenbroch (2002) compared student performance under no-choice fixed working schedules determined by the teacher versus choice working schedules (the students could determine their own schedules) and found that performance was better when students had to follow the no-choice fixed working schedules. If possible, a detailed syllabus including a “timetable” of the course, all deadlines, expected learning outcomes, and resources such as literature can help downsize the large degree of freedom of a study situation (cf. Eberly et al., 2001). Concerning the study program, an orientation event in the first semester or even each semester might support students in seeing the program’s inherent structure. One should not only focus on the contents of the program but also on the best way to run through the program. An individual twist to the orientation could be a short workshop in which each student is encouraged to plan her or his semester, thereby downsizing the large degree of freedom by establishing a unique structure which, ideally, should take into account all other activities they wish to make time for (e.g., sports, family, job), as well. Teaching styles that support student autonomy (Codina et al., 2018) may also be helpful. Finally, note that a large degree of freedom in the study situation is not alleviated by the introduction of more external control. Indeed, procrastination research demonstrates that external control is associated with increased procrastination (e.g., Janssen and Carton, 1999). We argue instead that unnecessary freedom should be reduced, as in the Ariely and Wertenbroch (2002) study discussed.

### Long Deadlines

#### Relevant Research

The idea of hyperbolic discounting helps to explain why we procrastinate the start of an activity. For example, according to the Temporal Motivation Theory (TMT; Steel and König, 2006; Gröpel and Steel, 2008), motivation increases as a function of the expectancy of an outcome and the size or value of a goal, but decreases as the time span before this outcome lengthens and impulsiveness increases. Thus, procrastination is more likely to occur if the outcome of an activity offers rewards in the distant future, and more so if impulsiveness is high (as is the case in procrastinators). Hence, immediate temptations often come to dominate over distant rewarding goals.

#### Relation to the Academic Environment

Results from qualitative (Schraw et al., 2007) and quantitative studies (Tice and Baumeister, 1997; Schouwenburg and Groenewoud, 2001) support the idea that the tendency to procrastinate decreases as the deadline for the task in question is approaching. Students find tentative due dates as especially frustrating (Schraw et al., 2007). In the absence of deadlines, students often set deadlines for themselves. Although such deadlines may work to reduce procrastination, they may actually reduce performance (Ariely and Wertenbroch, 2002). Other research, focusing on planning, has demonstrated that individuals tend to underestimate the necessary time it takes to complete tasks (the planning fallacy; Kahneman and Tversky, 1979; Kahneman and Lovallo, 1993) and to prefer longer deadlines when allowed to choose (Solomon and Rothblum, 1984). Recently, Zhu et al. (2019) demonstrated that long deadlines induce an inference of the focal task as more difficult, thereby making the student to allocate more time and resources to the task. However, the downside is that such elevated resource estimates may induce longer intention-action gaps (time before starting the task) and higher likelihood of quitting.

#### Remedies

While students with a broad range of self-management skills are able to deal with long and tentative deadline by breaking distant goals into nearer sub-goals themselves, students who lack these skills would benefit from structural arrangements defining sub-goals with timely deadlines. For instance, having students hand in an outline for a paper after the first third of the semester, the first draft after the second third, and the final draft at the end of the semester help to break a distant goal down to nearer sub-goals. Ideally, this scaffolding of self-regulating learning and writing might function as a model for future tasks with long deadlines. In general, making goals proximate (e.g., in the form of sub-goals) may help the student increase performance and reduce procrastination (e.g., Steel et al., 2018). Also, as reviewed by Gollwitzer and Sheeran (2006), adapting specific implementation intentions (“if-then”-plans rather than overall goal intentions) may have a strong effect on goal attainment. When students experience difficulties in goal striving, focusing on the main obstacle hindering progress is recommended (mental contrasting; e.g., Duckworth et al., 2011).

### Task Aversiveness

#### Relevant Research

Procrastination can be understood as a form of short-term mood-regulation (Sirois and Pychyl, 2013). Bad mood and negative feelings associated with a task is often repaired by avoiding the task and engaging in a pleasant task instead. The role of task aversiveness in triggering procrastination has received strong support (for a summary, see Steel, 2007). Closer examination of the task aversiveness literature demonstrates that aversive tasks are characterized by lower autonomy, lower task significance, boredom, resentment, frustration, and difficulty (Milgram et al., 1988; Milgram et al., 1995; Blunt and Pychyl, 2000; Steel, 2007). Moreover, Lay (1992) found that procrastinators tend to perceive common tasks in everyday life as more aversive compared to non-procrastinators, suggesting that procrastinators face the world with a negative bias toward task execution in general. As aversive conditions tend to motivate negatively by avoidance or escape, passivity is a likely effect (Veale, 2008). In sum, working under negative motivation is common in procrastinators, and a negative motivational regime is associated with passivity.

#### Relation to the Academic Environment

As study-related tasks typically are imposed by others (teachers, exams), they represent an important part of the academic environment for students. Such conditions are known to induce aversiveness and thereby procrastination. For example, when applying the Procrastination Assessment Scale-Students (Solomon and Rothblum, 1984), one prominent dimension turns out to be *aversiveness of task*. Time sampling as well as daily logs also show that the more students dislike a task, the more they procrastinate (Steel, 2007). Results of qualitative interview studies support these findings (Grunschel et al., 2013; Klingsieck et al., 2013; Visser et al., 2018).

Why students perceive academic tasks as aversive may be traced to the fact that students entering the university often lack adequate study skills to successfully managing mastery tasks^1^. Considering academic writing, for example, The Stanford Study of Writing indicates that, for most writers, the transition from high school to college writing is enormously challenging (Rogers, 2008). Moreover, university students report a variety of problems associated with academic writing (e.g., being aware of not being able to meet expected standards; Achieve Inc., 2005). In the last decades, universities have addressed the need for training academic writing by implementing writing centers. However, as discussed in another section (→Low focus on study skills training), instruction covering study skills is rarely provided. Thus, students often perceive academic tasks as aversive due to their lack of perceived competence. This effect may be amplified by low academic self-efficacy commonly seen in new students. Academic self-efficacy is negatively correlated to procrastination (*r* = −0.44; van Eerde, 2003), indicating that procrastinators perceive academic tasks as even more difficult (and therefore more aversive) compared to others. Indeed, a recent study^2^ found that students perceive academic tasks (e.g., present at a seminar) as more aversive compared to non-academic tasks (e.g., clean one’s apartment), but for both categories, aversiveness scores correlated positively with dispositional procrastination scores.

#### Remedies

The Self-Determination Theory (Deci and Ryan, 2002) suggests that tasks and conditions which meet a learner’s need for autonomy, competence, and relatedness support the internalization of extrinsic regulations and values, which in turn makes the task less aversive. Learners are more likely to internalize a learning goal if they embrace the meaningfulness or rationale of a task or activity if the underlying task or activity promotes their feeling of competence and if they are able to connect with other learners and experience a feeling of relatedness. Thus, formulating meaningful learning goals that lead to learning activities that fit the students’ competence level will make the task less aversive. Carefully crafted group tasks (→Inefficient group work) can also reduce procrastination. These kinds of tasks should foster the self-determination of learners. If one then embeds the learning activities in realistic learning settings, learners might even get interested in the learning activity. Game-based learning provides an innovative possibility for learning settings (Breuer and Bente, 2010). Finally, as discussed elsewhere (→Low focus on study skills training), programs for students entering the university should not shy away from offering training even in the most basic study skills.

### Temptations and Distractions

#### Relevant Research

Individuals are tuned toward attainment of positive outcomes and escape from or avoidance of aversive events. In procrastinators, this picture is exaggerated, with current attractive and aversive events dominating over distant ones. Procrastinators tend to be impulsive and present-biased (van Eerde, 2003; Steel, 2007), scoring high on scales measuring susceptibility to temptation, distractibility, and impulsivity (Steel et al., 2018). In fact, the correlation between distractibility and procrastination is very high, *r* = 0.64–0.72. Thus, procrastinators are especially vulnerable to environments with an abundance of temptations and distractors, as such environments tend to capture attention and divert planned behavior into more pleasurable activities available here and now. When working with aversive tasks (→Task aversiveness), this tendency increases, as the student will be motivated to escape the aversive situation as well as divert to something attractive (Tice et al., 2001).

#### Relation to the Academic Environment

Academic environments offer a large number of temptations and distraction, Internet access being a prime example (e.g., Reinecke and Hofmann, 2016). Mobile phones and laptops may have internet access everywhere on campus, presenting a continuous temptation and distractor, even during lectures. Universities tend to rely on web-based information and registration systems, and there is an increasing emphasis on digital utilities designed to assist learning, all necessitating continuous Internet access. The downside is that this situation presents a continuous challenge to students, especially those low in self-control (Panek, 2014). Internet use has often been shown to conflict with other goals and obligations (Quan-Haase and Young, 2010; Reinecke and Hofmann, 2016), and Lepp et al. (2015) demonstrated that total usage of mobile phones among undergraduates is negatively related to academic performance. Procrastination implies that the individual spends less time on focal tasks (Lay, 1992), and time spent on distracting tasks add to the problems procrastinators already experience. Internet multitasking (accessing the Internet while doing something else) is positively correlated with procrastination (Reinecke et al., 2018a,b), indicating that procrastinators are especially prone to suffer when Internet access remains unrestricted.

#### Remedies

Intervention studies (Hinsch and Sheldon, 2013) have demonstrated that reduction in leisure-related Internet use results in decreased procrastination and increased life satisfaction. Hence, limiting the availability of Internet use is a simple way of reducing these problems. Several companies practice restriction on use of mobile phones/laptops during meetings, and universities may consider similar measures. Universities may arrange wifi-free zones for teaching and studying, and teachers may ask students to turn off their laptops/phones during classes. For many, such advice may seem counterintuitive, as the use of “modern technology” in education is generally welcomed. However, given the detrimental effects associated with unrestricted Internet use seen in the part of the student population struggling with procrastination (i.e., half or more of all students), our advice is clear.

### Limited Information for Proper Self-Monitoring

#### Relevant Research

In self-regulated activities, three factors are particularly important for students (Baumeister and Heatherton, 1996): The student must have some standard to aim for (e.g., obtain a good grade in a course), monitor progress toward this standard, and correct as necessary if progress deviates from what is necessary to reach the standard. Although all three factors are important, Baumeister and Heatherton (1996, p. 56) pointed out that monitoring is crucial: “Over and over, we found that managing attention was the most common and often the most effective form of self-regulation and that attentional problems presaged a great many varieties of self-regulation failure.” As procrastination is considered a prime example of a self-regulation failure (Steel, 2007), it is likely that managing attention when working toward long-term goals is particularly vulnerable in procrastinators.

#### Relation to the Academic Environment

Due to the large degree of freedom in the study situation, the successful student needs information to keep an updated track of status, given long-term plans. Unfortunately, the study situation typically provides limited information. In many cases, exams (often held at the end of the semester) are the main source of feedback for students. Other kinds of information on progress (e.g., time spent at the university, participation in classes, observation of other students) may be unreliable as indicators of being on track. Furthermore, as consequences of procrastination are positive in the short term but not so in the longer term, learning is biased in favor of immediate positive consequences, and corrective action from long-term negative consequences is less likely.

#### Remedies

Measures that reflect goal-striving according to plan should be implemented. From the institutional/teacher perspective, such measures should focus on reading plans, course progress, and submissions, and should not be mixed up with study performance (e.g., grades). For example, as procrastination is a reliable predictor of study effort, high procrastinators spending less time in self-directed work (Lay, 1992; Svartdal et al., 2020), actual time spent on self-directed studying may be relevant information for many. Self-testing, recommended as an effective learning strategy (→Low focus on study skills training), also assists self-monitoring. Activity diaries, inspired by behavioral activation for depression interventions (e.g., Jacobson et al., 2001), may increase students’ awareness of how they spend their time as students. In recent years, several mobile apps have been developed to help students keep track of how they spend their time in the study situation (e.g., Dute et al., 2016), but little is known about the effect such apps may have in reducing procrastination.

### Low Focus on Study Skills Training

#### Relevant Research

In a qualitative study, Grunschel et al. (2013) found that students reported a lack of study skills as a notable reason for academic procrastination. One likely explanation is that low skills make tasks more effort demanding, and individuals are more likely to procrastinate on effort-demanding tasks (Milgram et al., 1988). Low academic skills also make academic tasks more frustrating, boring, and difficult, which are also factors reliably associated with task aversiveness (Blunt and Pychyl, 2000). As discussed in another section, task aversiveness is a reliable predictor for procrastination (→Task aversiveness).

#### Relation to the Academic Environment

A large part of academic work is spent on self-directed learning, and the skills needed to properly maneuver in such an environment is essential for student success (Kreber et al., 2005). Unfortunately, most students have not received instruction on effective and timely study skills (e.g., Dunlosky et al., 2013; Dunlosky and Rawson, 2015), and universities are slow in implementing effective skills instruction (Goffe and Kauper, 2014; Wieman and Gilbert, 2015). Teachers’ knowledge of effective study strategies is also lacking (Morehead et al., 2016; Blasiman et al., 2017).

#### Remedies

Study skill training programs produce beneficial effects in terms of academic performance and retention (Hattie et al., 1996; Gettinger and Seibert, 2002; Robbins et al., 2004; Wibrowski et al., 2017). Moreover, studies point out that learning how to study effectively cannot be separated from course contents and the process of learning (Weinstein et al., 2000; Durkin and Main, 2002; Wingate, 2007). That is, study skills training should be tailored for study programs or courses. They should suit the instructional context and teaching practices, expected achievement outcomes, and promote a high degree of learner activity. However, the impact of such skill learning interventions diminishes over time (Wibrowski et al., 2017), suggesting that repetition may be crucial. Thus, dedicating a portion of instruction time or having a study skill seminar at the beginning of each semester or course may be a good strategy. Different interventions may be considered depending on the course tasks (Schraw et al., 2007), students’ abilities and performance level (Hattie et al., 1996). Furthermore, as knowledge of study skills are not automatically translated into good study habits, academic self-efficacy (see next section) is important for circumventing procrastination (Klassen et al., 2008).

### Lack of Self-Efficacy-Building Opportunities

#### Relevant Research

Self-efficacy, our belief in our ability to manage a task, influences how willing we are to take on domain-specific challenges. The higher self-efficacy, the more likely we will take on a task (Bandura and Schunk, 1981). Even when ability to perform a task is high, but self-efficacy for that ability is low, the likelihood of prioritizing the task goes down, and procrastination is likely (Haycock et al., 1998; Klassen et al., 2008). Importantly, the relation between self-efficacy and procrastination is relatively strong and negative, *r* = −0.44 (van Eerde, 2003).

#### Relation to the Academic Environment

Self-efficacy is one of the strongest predictors of academic performance (Klomegah, 2007), yet is often neglected in course instruction. We have long known that students develop their self-efficacy for any academic task by gradually increasing proficiency with it (Bandura, 1997). Furthermore, as self-efficacy tends to be context-specific and will not automatically transfer over different tasks or activities (Zimmerman and Cleary, 2006), a relatively broad set of on efficacy-building experiences, course by course, is necessary (→Lack of study skill training), though not necessarily enough on its own (Kurtovic et al., 2019). Other research has recently indicated that self-efficacy may be indirectly rather than directly related to academic procrastination (Li et al., 2020), and that self-efficacy for self-regulation, for example, may be a strong predictor (Zhang et al., 2018).

#### Remedies

To improve self-efficacy, instructors can create more opportunities for mastery experiences by breaking down course assignments into manageable bits that are not too easy but still are possible for students to succeed at (Bandura, 1997), and by helping students self-reflect on their performance such that they feel more self-efficacious in the forethought phase of subsequent work (Zimmerman, 2000). As self-efficacy increases, and the likelihood of engaging in a task goes up (Ames, 1992), anxiety goes down (Haycock et al., 1998), establishing a virtuous circle of self-efficacy instead of a vicious circle of procrastination (Wäschle et al., 2014). This can be done through in-class activities or short assignments where the goal is to scaffold student learning with positive feedback and concrete information for how to improve on increasingly challenging versions of the task (Tuckman and Schouwenburg, 2004).

### Inefficient Group Work

#### Relevant Research

Students often work in groups (e.g., discussion groups, seminars), but often lack the basic skills for making group work effective. Group work also increases the probability of social loafing, the tendency for individuals to demonstrate less effort when working collectively than when working individually (Karau and Williams, 1993). Students may therefore often prefer to work alone as an alternative. However, working alone is associated with increased procrastination (Klingsieck et al., 2013). Qualitative evidence suggests that group work with interdependence between group members may reduce academic procrastination (Klingsieck et al., 2013). In support, results from educational psychology have shown positive effects of interdependent group work on individual effort in settings of cooperative learning. These studies also demonstrate beneficial effects of interdependence on social support, self-esteem, and health outcomes of group members (Johnson and Johnson, 2002, 2009). Taken together, these findings indicate the potential benefit of group work with interdependence, which may be harnessed in educational settings to reduce academic procrastination.

#### Relation to the Academic Environment

Although the beneficial effects of student group work in higher education seem evident (Springer et al., 1999; Johnson and Johnson, 2002), group work is neglected in curricula of many study programs, leading students to work individually on tasks and assignments and thus possibly promoting procrastination. Students in such programs may not always feel inclined to form study groups on their own and create more favorable group work conditions instead. This is especially unfortunate as methods and tools for group learning and studying abound.

#### Remedies

Group work with interdependence may be well suited to reduce procrastination among group members. Implementing group work with interdependence should be quite straightforward, for example by having groups work on projects or by adapting individual assignments to become interdependent tasks. The latter can be achieved by designing subtasks that need to be completed sequentially by assembling groups in such a way that each member contributes unique skills, or by formulating group-level goals and rewards (Weber and Hertel, 2007).

### Influence of Peers

#### Relevant Research

Prior research has indicated quite complex findings regarding the role of peers in facilitating or inhibiting procrastination (e.g., Nordby et al., 2017). Of the different ways in which peers may influence procrastination, three factors seem to be particularly important: social norms, observational learning, and distraction. Harris and Sutton (1983) suggested that an organization’s norms can either encourage or discourage procrastination, depending on whether norms suggest a prompt or delayed processing of tasks. Observational learning can support acquisition, inhibition, and triggering of many types of human behavior (Bandura, 1985), including procrastination. Thus, learning from others may also influence procrastination as well as strategies against it.

#### Relation to the Academic Environment

With regard to social norms, Ackerman and Gross (2005) found less procrastination among students when perceived norms suggested to start promptly. Social learning of procrastination or strategies against it have not been demonstrated empirically. However, on a more general level, observational learning has been shown to influence students’ self-regulatory skills (e.g., Zimmerman and Schunk, 2004). Indirect support for this notion also comes from Klingsieck et al. (2013) and Nordby et al. (2017), who report that peer behavior is taken into account by procrastinating students. With regard to social distraction, an early study reported peer influence to be a possible, yet not very frequent reason for procrastination (Solomon and Rothblum, 1984). Both qualitative (Klingsieck et al., 2013) and quantitative (Chen et al., 2016) evidence support the idea that distraction by peers can be a source of academic procrastination. A lack of social integration has also been reported an antecedent of academic procrastination (Patrzek et al., 2012), suggesting a balanced judgment on the role of peers and social contacts.

#### Remedies

Communication of social norms to start tasks promptly can occur through regular class instruction, thus supporting timely beginning of students with a disposition to procrastinate. Social cognitive theory predicts that social learning is facilitated, among others, by the salience of both model behavior and vicarious reinforcements (Bandura, 1985). Letting students reflect on and share their experiences with procrastination and strategies against it may support more productive observational learning.

## Discussion

This paper discusses nine factors characteristic of student study environments that, singly and in combination, increase the probability of procrastination. Clearly, given the high prevalence of academic procrastination, it is important to have an increased awareness of such risk factors and how they can be handled in order to prevent and reduce procrastination. Although we cannot control what students do, we can control how institutions encourage more productive behaviors for student success. We now briefly discuss how policymakers, universities, teachers, and students should approach these issues.

### Do the Factors Point to Common Problem Areas?

**FIGURE 1:**
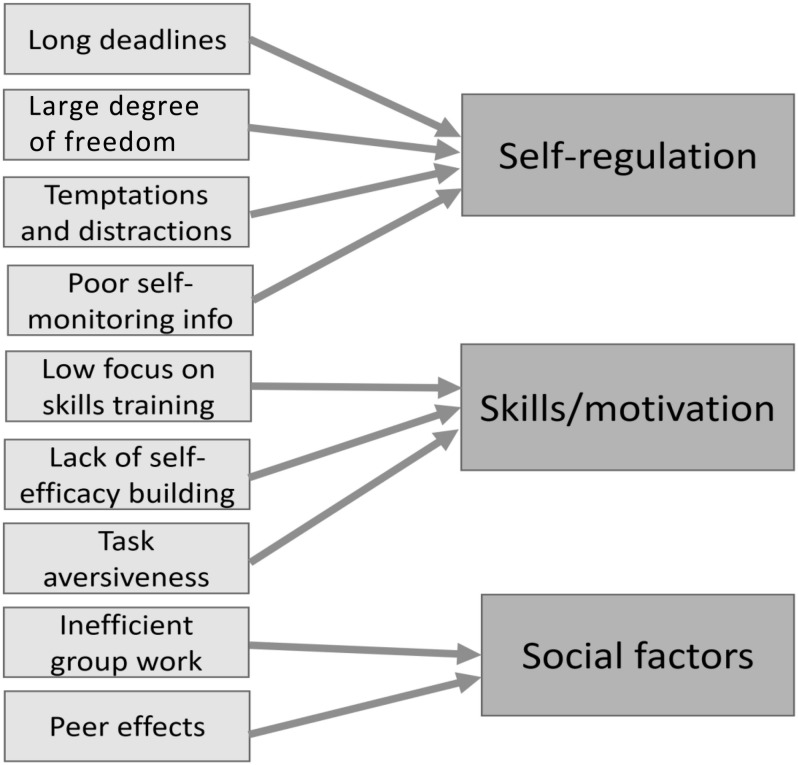
How procrastination-friendly factors relate to important themes in education.

Yes. We argue that the nine factors discussed can be loosely grouped into three themes (see Figure 1). First, four or five of the factors discussed (i.e., long deadlines, large degree of freedom in the study situation, temptations and distractions, poor self-monitoring information, and low focus on skills training), while being contextual and situational in nature, all relate directly to students’ ability to effectively self-regulate in the study situation. In effect, our overview indicates that the core problem of procrastination, poor self-regulation (Tice et al., 2001; Steel, 2007; Hagger et al., 2010), is amplified by common aspects of the student environment. An important implication of this insight is that training in self-regulation techniques among students (which we recommend) should not only be tailored to the specific needs of the students (cf. Valenzuela et al., 2020) but should also be supplemented with specific contextual and organizational measures that can support productive self-regulation. Since it is well known that self-regulation in the academic setting is important for performance (e.g., Duckworth and Seligman, 2005), it is paradoxical that academic institutions organize academic student life in ways counter to this insight.

Note that the problems in self-regulation seen in procrastination episodes may relate to skills factors (e.g., planning, monitoring), speaking for relevant skills training to strengthen self-regulation. However, often factors that undermine effective self-regulation are of primary importance in procrastination (e.g., Tice et al., 2001). For example, low energy and tiredness may render the individual more vulnerable to task-irrelevant temptations and distractions and increase task aversiveness, which in turn increases the probability of procrastination (Tice et al., 2001; Baumeister and Tierney, 2011). Insufficient sleep, common in the student population (e.g., Lund et al., 2010), is an important source of low energy and tiredness. Importantly, Knapstad et al. (2018) found that the most frequently reported health problem (as measured by the Somatic Symptoms Scale, SSS-8; Gierk et al., 2014) among a large sample of Norwegian students was a “Feeling of tiredness and low energy,” 45% of the students indicating that they were “fairly much or “very much” affected. This suggests that factors that undermine self-regulation among students should receive increased attention.

Second, the academic context can be designed to redress the skills and motivational issues that are often associated with procrastination. Low focus on study skills training and relative lack of efficacy-building opportunities represent a problematic combination that may themselves contribute to students perceiving academic tasks as aversive, thereby increasing the probability of procrastination. All these combined represent a disadvantageous motivational regime for academic work. The present overview identified specific organizational measures that institutions can take to change this situation. As discussed, increased focus on study skills training in concert with regular teaching may be a solution, as repeated mastery experiences will build self-efficacy as well as reduce task aversion.

Third, we should address the social factors that distract students from their academic work. By acknowledging that procrastination is a trap for students working alone, more opportunities can be made to encourage more collaborative work with others. It is important to carefully design group work in that it resembles interdependent group work. Furthermore, group work with student peers can be deliberately designed to increase student accountability, facilitating more need for self-regulation and offering students the opportunity to observe others with more productive self-regulation skills.

### Given the Large Number of Factors Discussed, Are Some Particularly Important?

We have not attempted to identify effect sizes to each of the variables discussed, and for many such estimates do not exist. Comparing the factors is, therefore, extremely difficult. Further, as several of the factors discussed have been linked to procrastination in correlational research, causality must be inferred with caution. Nevertheless, all the factors discussed have potentially large causal power to instigate and sustain procrastination. Overall, the factors examined focus on larger problem areas (i.e., self-regulation, skills and motivation, social factors), but each factor identifies concrete measures to be considered to implement changes.

In approaching such factors, all should ask: What can be changed on my part? Several of the factors (e.g., large degree of freedom in the study situation, long deadlines, temptations and distractions) address organizational and educational issues that should be addressed by organizations and teachers. Others (e.g., task aversiveness) imply more complex instructor-student interactions. For example, negative emotions in task aversiveness should be approached by teachers and students in cooperation by reducing task-associated risks and imbuing the tasks with personal relevance (van Grinsven and Tillema, 2006; Rowe et al., 2015), by enabling and encouraging student ownership of learning tasks (Rowe et al., 2015), and by facilitating frequent successful learning experiences that increase self-efficacy.

### Does It Make Sense to Implement Changes in One or Few Factors, Leaving Out Others?

Given an abundance of factors discussed, each capable of instigating procrastination, the high occurrence of procrastination in the student population is not at all surprising. Would it help, then, to change one or perhaps a few factors? One possible answer is that focusing on one factor is better than doing nothing. However, the downside of such an approach is that this single factor may not generate noticeable changes alone. Our recommendation would rather be to evaluate several or all factors and then implement changes as suitable within a single course, across courses, or in study programs. Note here that several of the factors discussed are relatively closely interwoven. For example, a large degree of freedom in the study situation often also implies long deadlines, suggesting that two factors may be addressed at once.

In such evaluations, it should be noted that each of the factors discussed is presented at a rather abstract level, so that relevance and concrete implementations in various settings must be carefully considered. For example, study topics vary by their very nature in how much freedom they represent for the student. Some study programs are already strictly structured and typically involve a common study group from start to finish, indicating that such programs do not need an increased focus on structure. Other programs are less structured and may also, by the nature of their study contents, be more “procrastination friendly” (e.g., Nordby et al., 2017). In other cases, such as study skills training and efficacy-building opportunities, “the more, the better” seems appropriate when closely linked to actual course learning tasks.

In evaluating the need for implementation of changes, the relevant factor should be assessed not only at the institutional level but—probably more importantly—at the program and course level. This applies not only to a need-based evaluation (“What do students need in order to reduce their procrastination?”), but also to a competence evaluation (“Can we provide the necessary work required for this implementation?”). Note also that some measures may be quite easy to plan on paper, but difficult to implement in a more complex system of rules and bureaucracy. For example, although long deadlines should be warned against (they induce procrastination), finding alternative solutions that can handle shorter deadline in a proper way may require changes (e.g., legal or practical) that are not easily possible to implement.

### Where to Start?

In developing prevention or interventions programs concerning procrastination, one has to keep the interplay between personal factors (i.e., student characteristics) and contextual factors (i.e., institutions, courses, and teachers) in mind. As can be seen from Table 2, the recommendations on the institutional, course, and teacher side will only fully unfold their effectiveness if students are simultaneously prepared to work on their self-regulatory skills. Thus, the recommendations we present in this paper should be accompanied by a culture of goal-focused self-regulation training programs. And, as discussed, self-regulation training programs, whether preventive or interventional, should not be administered without paying attention to contextual procrastination-friendly factors.

**TABLE 2:** Recommended measures to reduce procrastination.

Problem	Solution, institution/teacher perspective	Solution, student perspective
1.Large degree of freedom in the study situation	Restrict unnecessary choice; provide instruction on self-regulation for teachers to help students better self-regulate; create clearer frameworks for structuring course learning	Take course on self-regulation
2.Long deadlines	Implement short deadlines where possible; provide instruction on self-regulation for teachers to help students better self-regulate; create clearer frameworks for structuring course learning	Deliberately develop self-regulation skills for planning, monitoring, and controlling your learning
3.Task aversiveness	Formulate learning goals that students can make more personally meaningful; provide study skills instruction relevant for core tasks	Take courses on study skills; actively work throughout your studies on developing skills for how to make material personally relevant
4.Temptations and distractions	Limit unnecessary temptations and distractions	Beware of unnecessary temptations and distractions and work actively to develop skills that help you delay distractions until your planned academic work is done
5.Limited information for proper self-monitoring	Provide students with information on study progress; help students monitor their progress in goal-related activities	Increase your awareness of study progress, study habits, and how your spend your time; monitor your progress and identify when your strategies are insufficient; stop your use of inefficient strategies and replace them with more effective ones.
6.Low focus on study skills training	Provide study skills training for teachers as well as for students; link such training to course contents	Learn study skills that have been shown to be effective for effective learning—learn *how* to use them and, equally importantly, *when*.
7.Lack of self-efficacy- building opportunities	Provide learning opportunities with mastery experiences; provide concise and positive feedback	Arrange your learning to achieve many small successes: monitor those successes and reward yourself when you do well
8.Ineffective group work	Arrange interdependent study groups where each member is responsible for a unique task necessary for helping achieve the group goals	Participate in groups, ensuring that your role benefits the completion of group-level goals
9.Influence of peers	Establish explicit norms for academic work addressing timely engagement in academic tasks	Beware of the models you choose to learn from—chose those who perform as you would like to

## Conclusion

Given the high prevalence estimates of procrastination among students, a closer look at procrastination-friendly factors in the academic environment is clearly warranted. The present paper identifies nine such factors and provides suggestions on how they may be changed in order to understand, prevent, and reduce academic procrastination. Clearly, more research is needed in this area, both with regard to the factors themselves (how many are they?) as well as to their interplay and relative importance. Given the potential beneficial effects for students, institutions, and society, we conclude that researchers should pay increased attention to social, cultural, organizational, and contextual factors in their endeavors to understand academic procrastination.

## Author Contributions

FS initiated the project, wrote the introduction and discussion parts. All authors contributed at least one section each to the review and edited the complete draft.

## Conflict of Interest

The authors declare that the research was conducted in the absence of any commercial or financial relationships that could be construed as a potential conflict of interest.

## Footnotes

1 We use «study skills» in a broad sense, referring to skills needed on the part of the student to successfully master various aspects of study tasks (cf. Tressel et al., 2019).

2 Svartdal et al. (2020). Unpublished data.
